# Infectious Diseases Associated with Desert Dust Outbreaks: A Systematic Review

**DOI:** 10.3390/ijerph19116907

**Published:** 2022-06-05

**Authors:** Eleni Vergadi, Glykeria Rouva, Maria Angeli, Emmanouil Galanakis

**Affiliations:** Department of Paediatrics, School of Medicine, University of Crete, 71500 Heraklion, Greece; eleni.vergadi@uoc.gr (E.V.); kelrouva@gmail.com (G.R.); aggeli92@hotmail.com (M.A.)

**Keywords:** airborne pathogens, climate change, desert dust, ecosystem and public health, global warming, pollution, microorganisms, infectious diseases, human health

## Abstract

Background: Desert dust outbreaks and dust storms are the major source of particulate matter globally and pose a major threat to human health. We investigated the microorganisms transported with desert dust particles and evaluated their potential impact on human health. Methods: A systematic review of all reports on the association between non-anthropogenic desert dust pollution, dust microorganisms and human health is conducted. Results: In total, 51 articles were included in this review. The affected regions studied were Asia (32/51, 62.7%) followed by Europe (9/51, 17.6%), America (6/51, 11.8%), Africa (4/51, 7.8%) and Australia (1/51, 2.0%). The Sahara Desert was the most frequent source of dust, followed by Asian and American deserts. In 39/51 studies the dust-related microbiome was analyzed, while, in 12/51 reports, the association of desert dust with infectious disease outbreaks was examined. Pathogenic and opportunistic agents were isolated from dust in 24/39 (61.5%) and 29/39 (74.4%) of the studies, respectively. A significant association of dust events with infectious disease outbreaks was found in 10/12 (83.3%) reports. The infectious diseases that were mostly investigated with dust outbreaks were pneumonia, respiratory tract infections, COVID-19, pulmonary tuberculosis and coccidioidomycosis. Conclusions: Desert dust outbreaks are vehicles of a significant number of pathogenic or opportunistic microorganisms and limited data indicate an association between dust events and infectious disease outbreaks. Further research is required to strengthen the correlation between dust events and infectious diseases and subsequently guide preventive public health measures.

## 1. Introduction

Climate change is one of the biggest health threats facing humanity. Dust outbreaks are part of the climate effects that affect air quality and ecosystems in general [1,2]. Recently, extreme climate phenomena and global warming have affected the frequency of dust and sandstorms [1,3]. Dust pollution is becoming more intense globally, as dry conditions allow the expansion of arid lands, which are the main source of dust [1,3].

Dust contains particulate matter (PM) that includes sand and decaying soil particles, minerals, pollutants and bioaerosols [1,2,4]. Among all the sources of non-anthropogenic pollution, desert dust outbreaks are the major cause of increased PM concentrations in the atmosphere [2]. Dust particles remain airborne for several weeks and travel across thousands of kilometers away from their source region, crossing oceans and continents and globally increasing dust concentrations [2,5].

Dust airborne particles are categorized based on their size to those less than 10 μm (PM10), less than 2.5 μm (PM2.5) and ultrafine particles (UFP, less than 100 nm) [4]. These particles are inhaled and deposited in airways, causing health problems [4]. Adverse health effects are mostly associated with PM less than 2.5 μm, particularly UFΡ, as these are absorbed by bronchial epithelial cells and trigger inflammatory responses and oxidative stress, increasing the risk for pulmonary and cardiovascular diseases [4,5,6]. Indeed, there has been increasing evidence that acute and/or chronic exposure to dust PM is associated with pulmonary and cardiovascular problems, as well as increased morbidity and mortality [5,6,7].

It has been recently recognized that PM is a considerable source of microorganisms [8]. Airborne dust particles that contain microorganisms are reasonably concerning as they facilitate transport over long distances around the globe, altering airborne microbiome and facilitating the spread of infectious agents [6,8]. Since air microbiome can directly impact human and ecosystem health, there are concerns on the type of microorganisms that are transported and their potential effects on human health. In this study, we systematically reviewed the literature and assembled information on the potential pathogens that can be transported in the atmosphere with dust particles, their origin and their impact on human health.

## 2. Materials and Methods

### 2.1. Search Strategy and Selection Criteria

This study was conducted in accordance with the “Preferred Items for Systematic Reviews and Meta-Analyses” (PRISMA) guidelines [9]. A comprehensive search of Medline and Scopus databases was conducted, without language nor date/year restriction, to identify articles reporting on the association between non-anthropogenic pollution due to desert dust particles and infectious diseases. Various search strategies were used, opting for maximum sensitivity. The following algorithm was used: (desert dust) AND (bacteria OR fung * OR virus OR microorganism OR infection OR pathogen). The last search was updated on the 28th of May 2022. The title and abstract of the articles were screened for eligibility. Cited references were also reviewed to identify additional relevant studies. Two authors independently assessed full-text articles according to study selection criteria. In cases of unresolved disagreement during the screening process, a third author was consulted to reach consensus. Included studies were then reviewed in detail for data extraction.

All studies that examined the content of airborne microbial communities in desert dust particles or the association of desert dust and infectious diseases on humans or animals were included. Duplicate studies and studies in non-English language were excluded. Review articles, in vitro or animal experiments, studies reporting on effects in aqueous ecosystems or plants and studies without report on specific microorganisms were also excluded.

### 2.2. Data Extraction

From each eligible study, data extraction was performed in a standardized electronic form. Information on authors, year of publication, time and duration of the study, region affected, dust source, type of microorganisms detected in dust particles and associated infectious diseases, if any, were recorded.

## 3. Results

### 3.1. Search Results and Included Studies

**Figure 1:**
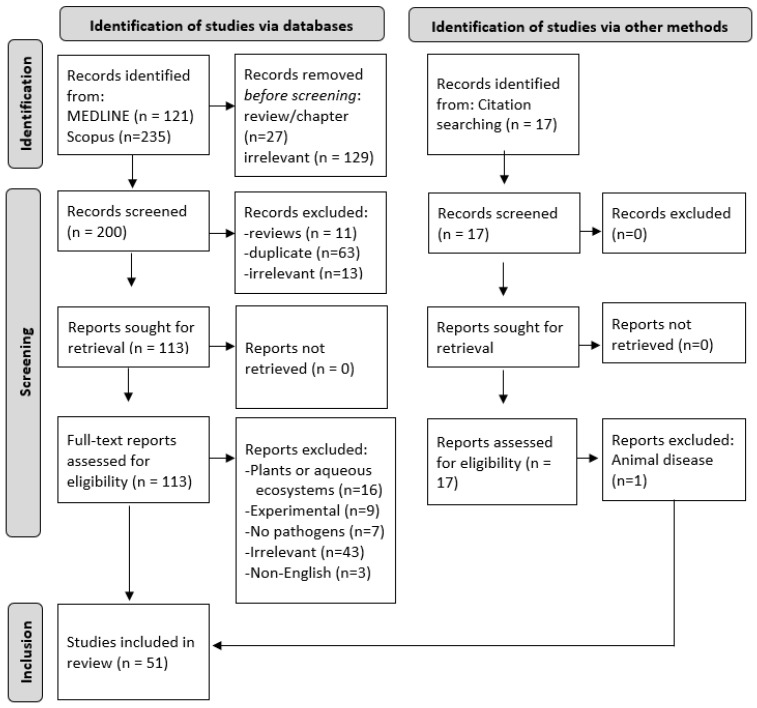
PRISMA flow diagram of study selection. PRISMA, Preferred Reporting Items for Systematic Reviews and Meta-Analysis.

Figure 1 illustrates the PRISMA flowchart of study screening and selection. A total of 356 records were obtained from database searches, and 17 records were identified from citation searches. Finally, 51 studies were included in this review after the removal of review articles (*n* = 38), duplicate articles (*n* = 63), irrelevant studies (*n* = 185), articles in non-English language (*n* = 3) or studies that did not fulfill inclusion/exclusion criteria (*n* = 33) (Figure 1).

### 3.2. Characteristics of Included Studies

All studies were published between 2004 and 2021 [10,11,12,13,14,15,16,17,18,19,20,21,22,23,24,25,26,27,28,29,30,31,32,33,34,35,36,37,38,39,40,41,42,43,44,45,46,47,48,49,50,51,52,53,54,55,56,57,58,59,60]. The affected regions that were studied were Asia (32/51, 62.7%), followed by Europe (9/51, 17.6%), America (6/51, 11.8%), Africa (4/51, 7.8%) and Australia (1/51, 2.0%). Among all included studies, 39/51 studies analyzed environmental samples and alteration in air microbiome, while in 12/51 studies, an association with dust storms and infectious diseases was performed. In experimental studies, the microorganisms were examined in air/aerosol samples (21/39, 53.8%), dust samples (10/30, 25.6%), soil/sand (6/39, 15.4%), or snow (2/39, 5.1%), collected after various dust storms in the period from 2004 to 2020. The Asian deserts were the more frequent source of dust in the included studies (22/51, 43.1%), followed by the Sahara/North Africa deserts (21/51, 41.2%), American deserts (6/51, 11.8%) and Arabian deserts (4/51, 7.8%).

### 3.3. Identified Microorganisms from Airborne Sampling

Isolated microorganisms with the potential to induce infections in humans are summarized in Table 1. Other microorganisms that have not been associated with human disease are summarized in Supplemental Table S1.

Pathogenic and opportunistic agents were isolated from dust-related microbiome in 24/39 (61.5%) and 29/39 (74.4%) of studies, respectively. The pathogenic microorganisms that were isolated in dust microbiome were *S. aureus*, *Streptococcus* spp., *N. meningitidis*, *Enterobacteriaceae* spp., *P. aeruginosa*, *Brucella* spp. and *Coxiela burnetti*, as well as *Coccidioides* spp. fungus and influenza A virus [10,16,22,25,26,27,28,29,30,31,32,33,34,35,36,37,38,39,40,41,42,43,44,45,46,47,48,49]. The remaining studies reported opportunistic pathogens, mostly *Bacillus* spp., *Aspergillus* spp., etc.

### 3.4. Associated Infectious Diseases

Among the included studies, 12 examined the association of dust storms with infectious diseases (Table 2). A significant positive association was found in 10/12 (83.3%) of them. The infectious diseases positively associated with desert dust storms were mostly pneumonia and other respiratory tract infections, including COVID-19, pulmonary tuberculosis and coccidioidomycosis (Valley fever) [11,13,17,18,19,20,23,24,60,61]. Other infectious diseases that have been positively associated with dust storms are measles and meningococcal meningitis [12,21]. In two studies, no significant link was noted between dust storms and the infectious diseases studied, pneumonia [13] or coccidiomycosis [11].

## 4. Discussion

Over the last decades, desert dust outbreaks and sandstorms have been associated with an increase in the concentration of microorganisms in the atmosphere and with several disease outbreaks [4,6,12]. In the present study, we systematically appraised the available evidence to better characterize the microorganisms with pathogenic potential that can be transported with desert dust particles as well as the association of desert dust events with infectious disease outbreaks. Based on our findings, among the studied regions, the most affected ones were Europe and Asia and most of the dust—related microbes originate from large deserts, particularly Asian deserts and the Sahara. In case of the Sahara, these data are in accordance with the fact that approximately more than 100 tons of dust travel each year from the Sahara throughout the globe [6].

Dust events are characterized by significant alternations in the air microbiome [10]. Several of these dust-derived microorganisms are environmental microbes that are highly resistant to stress and capable of surviving under harsh environmental conditions, such as UV radiation, extreme temperatures, drought and a lack of nutrients [61,62]. These microorganisms are expected to preserve the potential to proliferate once they are transported in more friendly environmental conditions [62]. However, this is not always the case, as in several studies, microbes sensitive to extreme environmental conditions have been identified [26,38,57]. There is indeed considerable variation in the type of microorganisms detected in the included studies, as a result of the differences in study design. For example, the included studies differed substantially on the regions studied, the sampling methods and microbiological analysis used, which are issues that may severely affect the microbiome isolated. Additionally, several studies were focused on particular pathogens only, such as fungi or influenza A [33,50]. A better understanding of the factors influencing the detection and composition of these microbial communities is important to address questions related to the impart of desert events on infectious diseases.

Interestingly, apart from the environmental microorganisms, in the great majority of studies, either pathogenic or potentially pathogenic microorganisms were detected. Many of these pathogens, such as *N. meningitidis*, *S. aureus*, *Enterobacteriaceae* spp., *Brucella* spp., *Coxiela burnetti* and *P. aeruginosa* are major human pathogens that may pose an important human health risk. However, data from the studies included in this review may allow us to make only indirect associations with infectious diseases as no direct link was investigated. Notably, the fact that pathogens such as *Brucella* spp., *Coxiella burnetti* and *Salmonella* spp. have been detected in the samples raises the possibility of the existence of herds of domestic animals in the pathway of dust to the sampling site. Furthermore, it is also possible that desert dust may affect the health of animals as well as the number of animal reservoirs in the affected regions. However, further studies are required to investigate the impact of desert dust in animal health.

As far as means of transmission and distance are concerned, potential human pathogens were found in all size fractions of dust tested, including the smaller particles that are more likely to undergo long-distance transport and reach the distal airways via inhalation [16]. In addition, some of the isolated pathogens, such as *Coxiella burnetti*, are well-known for their ability to be transmitted via inhalation from long distances. Airborne particles may not only affect human health through inhalation; they may also end up in soil and water with rain and, thus, affect plants, animals and the entire ecosystem. Transmission via fomites or via expansion of animal reservoirs may also be plausible, yet it remains unproven. 

While epidemiological studies over the last years have demonstrated that dust outbreaks are associated with severe respiratory and cardiovascular disease and increased risk of hospitalization and mortality, limited information exists on the role in promoting infectious disease outbreaks [63,64]. Based on our findings, limited data point towards a significant association of dust storms to infectious disease outbreaks. The most common infectious diseases associated with dust storms were respiratory tract infections, such as pneumonia, COVID-19, pulmonary tuberculosis, coccidioidomycosis and others. It is interesting that a potential association was also found with measles that, similarly to *M. tuberculosis*, is known to be transmitted via airborne aerosols [19]. In another study, Respiratory Syncytial virus infection has been associated with increased PM concentrations and haze; however, no data exist on the source of the haze; thus, correlations with desert dust cannot be made [65].

Interestingly, in another study, an increase in hospital admissions for meningococcal meningitis was noted in Barcelona one month after a Saharan dust intrusion [21]. Additionally, *N. meningitidis* was isolated in dust storm samples in several other experimental studies [22]. While other studies have also suggested an association of meningitis episodes with dust storms periods [66,67], it is not clear whether this association is direct or indirect. Based on Tobias et al., the incubation period for meningococcal meningitis is usually less than one week and it seems unlikely that a relationship with a delay of one month after a Saharan dust storm was direct [21]. In this case, dust events are likely to be correlated with other factors, such as changes in human behavior or the breach of protective mucosal barriers due to dryness that may then influence the risk of meningitis [21]. Indeed, we hypothesize that dust intrusion may affect the transmission of infectious diseases in an indirect way, via, for example, fomites for human pathogens, via expanding the animal reservoir (in case of zoonoses) or via affecting human behavior and/or the susceptibility of the host. Similar explanations are probably the case for the transmission of COVID-19, measles and coccidiomycosis in correlation with dust events. 

## 5. Conclusions

To conclude, it appears that desert dust events and sandstorms are vehicles of a significant number of pathogenic or potentially pathogenic microorganisms. Data on their association to infectious disease outbreaks are limited but are suggestive, in general, of a positive association. Considering that climate change and global warming are expected to increase dust activities globally, more research is needed to investigate viability and pathogenic potential of dust microorganisms and assess the risk for human health. This information is crucial to properly guide preventive public health measures to develop a better strategy for the preparation, prevention and mitigation of the destructive effects of these events.

## Figures and Tables

**Table 1:** Pathogenic and opportunistic microorganisms transmitted via desert dust outbreaks globally.

Region; Study	Dust Source	Sample	Potential Pathogenic or Opportunistic Microorganism
**Africa**			
Chad, Cape Verde Islands; [25]	The Sahara	dust samples	Firmicutes-Bacillacea, Proteobacteria-Oxalobacteraceae, Microsporidia
Mali; [16]	The Sahara	atmospheric particle samples	B. cereus, E. coli, P. aeruginosa, F. nucleatum
Mali; [27]	The Sahara	air particles	*Acinetobacter* spp., *Bacillus* spp., *Corynebacterium* spp., *Staphylococcus* spp., *Aspergillus* spp.
Senegal; [26]	The Sahara	dust samples	*Micrococcus* spp., *Bacillus* spp., *Kytococcus* spp., *Pseudomonas* spp., *Burkholderia* spp., *Brucella* spp., *S. aureus*, *Rhizobium radiobacter*, *Sphingomonas paucimibilis*, *Serratia plymuthica*, *Enterobacter cloacae*, *Aeromonas hydrophila*, *Serratia rubidaea*
**America**			
Chile; [28]	Atacama Desert	dust samples	*Kocuria flava*, *Bacillus subtilis*, *Brachybacterium paraconglomeratum*, *Oceanobacillus oncorhynchi*, *Microbacterium barkeri*, *Bacillus* sp., *Microbacterium paraoxydans*, *Bacillus firmus*, *Aspergillus versicolor*, *Aspergillus nidulans*
Mexico and New Mexico USA; [29]	Chihuahuan Desert	air (particular matter) and soil samples	*Fusarium* spp., *Aspergillus* spp.
***Asia***			
China, South Korea; [30]	Asian desert dust	sand samples	*Massilia* spp., *Planococcus* spp.
Dunhuang, China; [51]	Taklimakan Desert	airborne dust	*Staphylococcus* spp., *Pseudomonas* spp.
Dunhuang, China; [52]	Asian desert dust, Gobi desert	bioaerosoles	*Bacillus* spp., *Staphylococcus* spp.
China; [43]	Asian desert dust	air samples	Proteobacteria, Firmicutes, *Bacillus* spp.
South Korea; [37]	Asian desert dust	air samples	Prevotellaceae bacterium sp.
South Korea; [31]	Asian desert dust	air samples	Proteobacteria, Firmicutes
South Korea; [32]	Asian desert dust	air samples	*Bacillus* spp., *Bacillus circulans*; *Sphingomonas starnbergensis*
Korea; [44]	Asian desert dust	dust particulate matter	*Bacillus subtilis*
Korea; [45]	Asian desert dust	soil samples	*Staphylococcus* spp., *Bacillus cereus*
Taiwan; [33]	Asian desert dust	air samples	Influenza A virus
Taiwan; [50]	Asian desert dust	spore trap	*Penicillium* spp., *Aspergillus* spp.
Israel; [34]	The Sahara, Arabian deserts	aerosols	*Enterobacteriaceae* spp., *Lactobacillus* spp., *Corynebacterium* spp.
Israel; [36]	South Europe, North Africa	dust samples	α-Proteobacteria, Actinobacteria, β-Proteobacteria, Tremellomycetes
Israel; [49]	The Sahara	dust particles	*Aspergillus fumigatus*, *A. niger*, *Penicillium chrysogenum*
Lebanon; [35]	North African and Asian desert dust	dust rain samples	β-Proteobacteria, a-proteobacteria, Firmicutes, E-proteobacteria, γ-Proteobacteria
Japan; [47]	Asian desert dust	aerosole samples	α-Proteobacteria, β-Proteobacteria and γ-Proteobacteria
Japan; [39]	Asian desert dust	bioaerosols	*Bacillus subtilis*
Japan; [40]	Asian desert dust	air samples	Firmicutes (*B. subtilis*, *B. pumilus*), a-Proteobacteria
Japan; [53]	Gobi desert	dust samples	*B. subtilis* and *B. licheniformis*
Mongolia; [10]	Gobi Desert	air samples	α- Proteobacteria, β-Proteobacteria and γ-Proteobacteria
Mongolia; [48]	Gobi desert	soil samples	*Bacillus* spp., *Staphylococcus* spp.
Eastern Mediterranean; [41]	The Sahara Desert	air samples (particulate matter)	*Micrococcus terreus*
Iran; [42]	Arabian Deserts	air samples	*Bacillus* spp., Mycosporium
Kuwait and Iraq; [38]	Arid areas of Iraq and Kuwait	dust and soil samples	*Mycobacterium* spp., *Brucella* spp., *Coxiella burnetii*, *Clostridium perfringens*, *Bacillus* spp.
Iraq; [46]	Middle Eastern desert dust	air samples	*Bacillus* spp., *Micrococcus* spp., *Streptomyces* spp., *Staphylococcus* spp.
**Europe**			
Spain; [54]	The Sahara Desert	soil samples	Alpha- and Betaproteobacteria, Actinobacteria, Bacteroidetes, Firmicutes
Greece; [56]	The Sahara Desert	air samples	Firmicutes
Swiss Alps; [22]	The Sahara Desert	snow samples	Oxalobacteriaceae, *Neisseria* spp., *Streptococcus* spp.
Italy; [57]	African desert dust	air samples (particulate matter)	*Sphingobacterium multivorum*, *Clostridium cadaveris*, *S. aureus*, *Propionibacterium avidum*, *Propionibacterium acnes*, *Salmonella enterica*, *Providencia rettgeri*, *Acinetobacter lwoffi*, *Acinetobacter ursingii*, *Acinetobacter johnsonii*, *Enterobacter cloacae*, *Enterobacter asburiae*, *Enterobacter aerogenes*, *Enterobacter amnigenus*, *Enterobacter hormaechei*
Italy; [58]	The Sahara Desert	snow samples	*Bacillus* spp., Aurebasidium, Periconia, Pleosporaceae
Italy; [55]	African desert dust	dust samples	*Bacillus* spp., *Streptococcus* spp., *Lactococcus* spp., *Corynebacterium* spp., *Brevundimonas* spp., *Paracoccus* spp., *Sphingomonas* spp., *Aspergillus* spp.
**Australia**			
Australia; [59]	Australian desert dust	dust and rain samples	*Bacillus* spp., *Pseudomonas* spp.

**Table 2:** Desert dust events associated with infectious disease outbreaks.

Study	Region	Pathogens Studied	Association with Infectious Diseases
Bell, M.L., et al. [13]	Taiwan	-	Pneumonia (no significant association)
Cheng, M.F., et al. [60]	Taiwan		Pneumonia
Comrie, A.C., et al. [11]	California, USA	*Coccidioides* spp.	Coccidioidomycosis (Valley fever) (no significant association)
Kang, J.H., et al. [61]	Taiwan	-	Pneumonia
Lauer, A., et al. [15]	California, USA	*Coccidioides* spp.	Coccidioidomycosis (Valley fever)
Lauer, A., et al. [17]	California, USA	*Coccidioides* spp.	Coccidioidomycosis (Valley fever)
Ma, Y., et al. [12]	China	Measles virus	Measles
Rohrer, M., et al. [19]	Spain	SARS-CoV-2	COVID-19
Tobías, A., et al. [21]	Spain	*Neisseria meningitidis*	Meningococcal meningitis
Tong, D.Q., et al. [18]	Southwestern USA	*C. immitis*, *C. posadasii*	Valley fever
Trianti, S.M., et al. [19]	Greece	-	Pneumonia, other respiratory tract infections
Wang, Y., et al. [23]	China	*M. tuberculosis*	Pulmonary tuberculosis

## Supplementary Materials

The following supporting information can be downloaded at: https://www.mdpi.com/article/10.3390/ijerph19116907/s1, Table S1: Other microorganisms found in dust storms globally.
