# Supplementary material for: Infectious Diseases Associated with Desert Dust Outbreaks: A Systematic Review
Source: Int J Environ Res Public Health. 2022 Jun 5;19(11):6907. doi: 10.3390/ijerph19116907 (PMC9180817; doi:10.3390/ijerph19116907)
Supplement: Supplementary file 1 [file ijerph-19-06907-s001.zip › ijerph-1720280-supplementary.pdf]

Table S1. Other microorganisms found in dust storms globally.

Study/Continent	Region	Dust Source	Potential Pathogen
Africa			
Favet, J, et al. [25]	Chad, Cape Verde Islands	The Sahara Desert	Actinobacteria- Geodermatophilaceae, Nocardiodaceae, Solirubrobacteraceae, Rhizobiales, Sphingomonadaceae acteroidetes, Cytophagaceae<br>Ascomycota, Basidiomycota; Chytridiomycota, Glomeromycota
Kellogg, CA, et al. [27]	Mali; [27]	The Sahara	*Alternaria* spp,
America		gal για	
Azua-Bustos, A, et al. [28]	Chile	Atacama Desert	*Bacillus simplex, Bacillus litoralis, Bhargavaea cecembensis, Staphylococcus equorum, Solibacillus sivestris, Paenibacillus, Salinicoccus roseus, Bacillus amyloliquefaciens, Bacillus altitudinis, Arthrobacter sp, Terribacillus saccharophilus, Bacillus paralicheniformis, Ophiosphaerella herpotricha, Chaetomium globosum, Cladosporium bruhnei, Penicillium chrysogenum*
González-Delgado, A, et al. [29]	Mexico and New Mexico USA	Chihuahuan Desert	*Alternaria* spp, *Penicillium* spp
Asia			
An, S, et al. [30]	China South Korea	Asian desert dust	*Carnobacterium* spp, *Planomicrobium* spp, *Pontibacter* spp, *Pedobacter* spp, *Lysobacter anguibacter, Ohtaekwangia* spp
Cha, S, et al. [31]	South Korea	Asian desert dust	*Deinococcus Thermus*, Acidobacteria, Gemmatimonadetes and Chloroflexi
Cha, S, et al. [32]	South Korea	Asian desert dust	*Modestobacter* spp, *Methylobacterium iners*
Gat, D, et al. [24]	Israel	The Sahara Desert, Arabian Desert	Deinococcus Thermus, Chloroflexi, Cyanobacteria, Gemmatimonadetes, Acidobacteria
Gat, D, et al. [34]	Israel	The Sahara and the Arabian deserts	*Rombutsia* spp, *Jeotcalycoccus* spp
Itani, G.N, et al. [35]	Lebanon	North African and Asian desert dust	Actinobacteria, Bacteroidetes, Cyanobacteria, Dothideomycetes, Eurotiomycetes and Sordariomycetes
Katra, I, et al. [36]	Israel	South Europe, North Africa	Actinobacteria, Dothideomycetes, Mitosporic
Lee, S, et al. [37]	South Korea	Asian desert dust	Aquabacterium sp., Flavobacteriales bacterium,
Maki, T, et al. [40]	Japan	Asian desert dust	Cyanobacteria (Synechococcus spp.)
Maki, T, et al. [10]	Mongolia	Gobi Desert	Acidobacteria, Actinobacteria, Bacteroidetes, Chloroflexi,
Mazar, Y, et al. [41]	Eastern Mediterranean	The Sahara Desert	*Rubellimicrobium roseum;*
Yamaguchi, N, et al. [43]	China	Asian desert dust	Bacteroidetes, Actinobacteria, *Rubellimicrobium spp, Rubellimicrobium mesophilum, R. roseum*
Europe			
Barberán, A, et al. [54]	Spain	The Sahara Desert	Actinobacteria, Bacteroidetes
Roselli, R, et al. [55]	Italy	African desert dust	*Anoxybacillus* spp, *Methylobacterium* spp, *Caulobacter* spp

Meola, M, et al. [22]	Swiss Alps	The Sahara Desert	Gemmatimonadetes, Deinococcus Thermus, Flavobacteriaceae, Chloroflexi, Cytophagaceae, Comamonadaceae, Myxococcales, *Janthinobacterium* spp
Romano, S, et al. [57]	Italy	African desert dust	*Johnsonella ignava*, *Clavibacter michiganensis, Curtobacterium flaccumfaciens*
Weil, T, et al. [58]	Italy	The Sahara Desert	*Geodermatophilus* spp, *Deinococcus* spp, *Gemmatimonas* spp, *Arthrobacter* spp, *Nocardiodes* spp, *Rubrobacter* spp, *Solirubrobacter* spp Montagnulaceae, Embellisia, and Davidiella
